# Torsional shear strength of steel joined with high performance aerospace adhesives at cryogenic and elevated temperatures

**DOI:** 10.1371/journal.pone.0206981

**Published:** 2018-11-07

**Authors:** Gurdial Blugan, Gustavo Mata-Osoro, Simon Fecht, Jolanta Janczak-Rusch, Jakob Kuebler

**Affiliations:** 1 Laboratory for High Performance Ceramics, Empa, Swiss Federal Laboratories for Materials Science and Technology, Duebendorf, Switzerland; 2 Department of Adhesive Bonding Technology, Fraunhofer Institute for Manufacturing Technology and Advanced Materials IFAM, Bremen, Germany; 3 Laboratory for Joining Technologies and Corrosion, Empa, Swiss Federal Laboratories for Materials Science and Technology, Duebendorf, Switzerland; University of Vigo, SPAIN

## Abstract

Pure torsional shear tests of joints glued with two different aerospace grade adhesives were performed using a specifically designed and constructed torsional shear test equipment. The developed test equipment allows for measuring of pure torsional shear strength under cryogenic and at elevated temperature conditions. The adhesives Hysol EA 9321 and 3M Scotch-Weld EC-9323-2 B/A were used to join steel torsional shear test specimens. Torsional shear tests were performed from -180°C to 150°C. In addition torsional shear fatigue tests were also performed at various loads and the effect of cryogenic aging (cyclic cooling and warming) on the torsional strength of the joints was investigated. The results showed that both sets of adhesive joints achieved three times higher torsional shear strength at -180°C compared to room temperature.

## Introduction

Joining of materials is becoming a field of increased importance as more and more complex engineering solutions are found to today’s challenges. Advances in adhesive technologies in the last decade have led to a significant increase in the use of aluminium and composite materials in lightweight structures, especially in the automotive and aerospace sectors, which are not always easy to join by other technologies, e.g. riveting, brazing and soldering [1–3]. Epoxy adhesive systems based on two component thixotropic pastes are used for high strength joints in a wide range of structural components of metal, glass, plastic and ceramic to each other. A recent review of the properties of these structural adhesives was made by Pethick [4]. As more complex high performance structures are being manufactured using this type of adhesive, it becomes more important to characterize joints accurately and over a wide application temperature range. In the automotive sector typical vehicle testing is performed from -40°C to 80°C [5], whilst in the aerospace sector the desire is to have more accurate mechanical data of adhesive joints over an even greater temperature range. Two high performance adhesive systems which are widely used include Hysol EA 9321 and 3M Scotch-Weld EC-9323-2 B/A, both are used in the aerospace industry. The applications for these two systems also include use in space vehicles and satellites under cryogenic temperature conditions.

One of the most important mechanical properties measured for adhesive bonds is shear strength. Typical shear strength values which are available for these adhesives are based on the single lap shear test (e.g. EN 2243–1) and the tensile lap shear test (e.g. ASTM D1002). These lap shear based tests are apparent shear tests (i.e. technological tests), as the stress is a non-constant mix of shear and normal stresses (which can also be compressive or tensile in nature) both which vary in distribution over the specimen and joint geometry [6]. In addition gripping of specimens, frictional effects and transferring of load to the specimen can all affect the apparent shear strength values obtained. This can result in components of both shear and peel stresses in the measured apparent shear strength. There is therefore a fundamental need for more accurate shear strength data for designing of new components in these demanding environmental conditions. Unfortunately, many designers and engineers for reasons including historical, cost, ease of specimen manufacture, etc., still use apparent shear strength data based on technology tests (e.g. single lap offset shear test) instead of measuring pure shear strength.

Test methods which measure only pure shear are the torsional test and asymmetrical four point bending test (for ceramic joints). Theoretically the torsional shear strength test is also free of stress concentrations. A number of different designs of torsional shear test specimens have been evaluated in the literature with and without central holes and in different materials (materials with both plastic and brittle deformation), as well as with different joining materials [7–9]. Square section samples although easier to clamp and to manufacture can however lead to normal stresses and stress concentrations especially in brittle materials (e.g. ceramics) leading to catastrophic failure in the clamping region. On the other hand square joining sections can promote stress concentrations at the edges resulting in an apparent shear test. Therefore, a circular cross-section of the joint area with a square clamping zone as in a hour glass is often the preferred solution [7]. However, in practice this is not always suitable for all types of material systems, e.g. for C/C fibre composites as shown by Ferraris et. al. [6]. Another torsional shear test geometry is the napkin ring set up, where it can be difficult to produce test pieces especially with viscous adhesives where the adhesive will not stay in the joint [10]. Cylindrical section samples are also difficult to grip in a torsion test machine and hence to apply a uniform stress.

In addition these tests were all originally developed for room temperature measurements and not for elevated or cryogenic temperatures, which is especially important for aeronautical and space applications. To date there is very limited published data on the mechanical behaviour of adhesively bonded metal joints under cryogenic conditions, we have found only one article on shear strength (double lap joint), with one other on the fracture toughness of a composite joint [11, 12].

In the current work we perform torsional shear tests using steel samples joined with the aforementioned two high performance commercial adhesives over the temperature range of -180°C to 150°C in a specially designed and built torsional shear test jig. Steel is used as it is a model non brittle material which allows the preparation of a large number of samples in a reasonable time scale. Details of the torsional test jig, the sample geometries, the shear test conditions and the failure mechanisms over the temperature range are presented. Apart from the stress strain behaviour of the adhesive as a simple failure criterion, adhesives in joints also present problems with their long term behaviour. Therefore, to investigate the long term behaviour we performed room temperature cyclic torsional fatigue testing with glued joints at stresses below the torsional shear failure strength as well as measuring the shear strength on thermal cryogenically aged joints using the same torsional test equipment. Showing the versatility of the torsional shear setup and the possibility to obtain more accurate data with this test equipment which may be used for design and engineering of new joined components.

## Materials and methods

**Fig 1:**
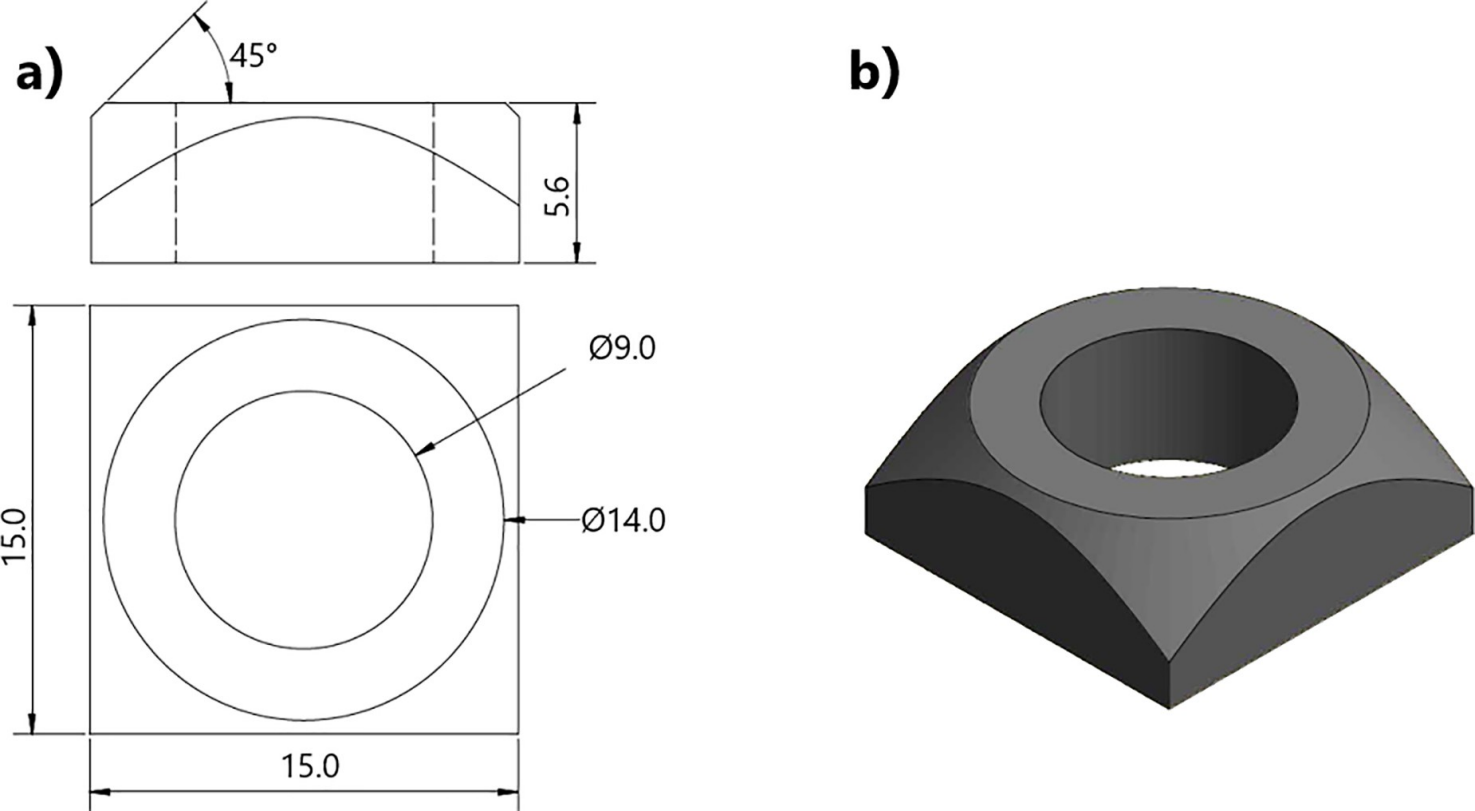
a) Design of the torsional shear test specimens manufactured in steel for testing of the adhesives b) 3-d image.

Type S235 steel (with a Young’s modulus of 210 GPa and a yield stress of 235 MPa) specimens with a square section for easy clamping and with cylindrical cross-sectional ring joint areas which are less susceptible to stress concentrations on edges and corners were manufactured. The design of the steel samples is illustrated in Fig 1. With a square section of 15 mm, the cylindrical ring area to be joined has an outer diameter and inner diameter of 14 and 9 mm, respectively. Although we tested a range of different designs of different dimensions, with and without holes, the design with a hole was used as it also allows testing with accurate temperature measurement.

**Fig 2:**
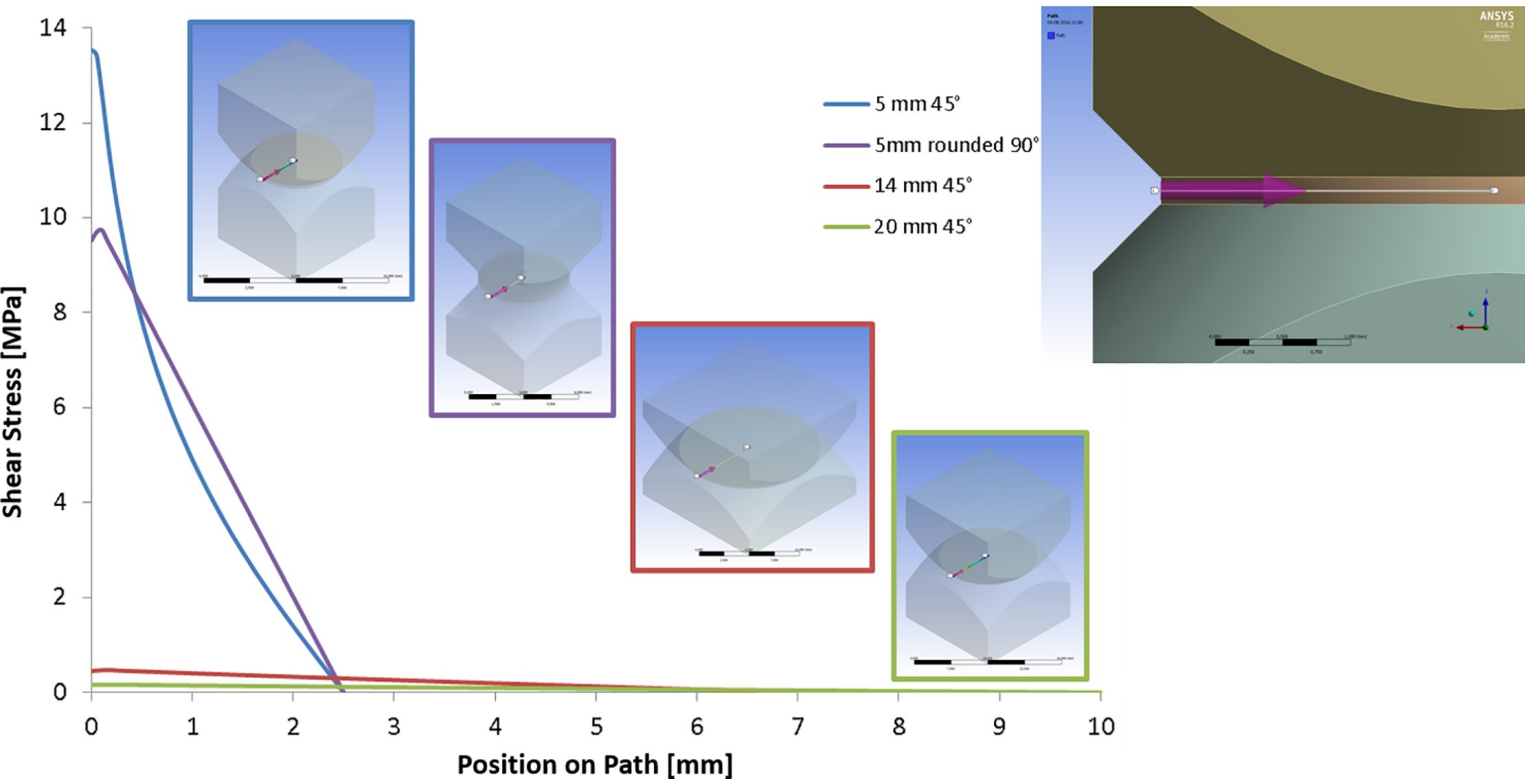
FE results of different samples with different dimensions and shape, showing the torsion shear stress in join as a function of distance from the outer surface.

**Fig 3:**
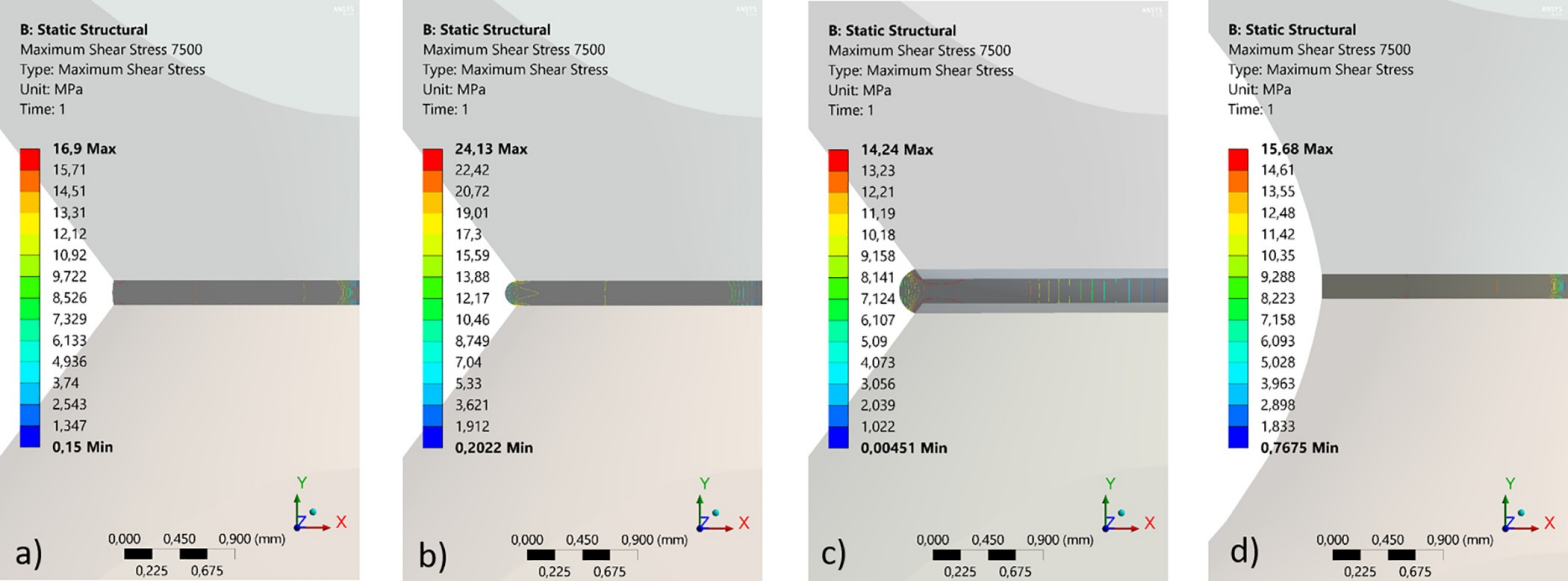
**FE results of shear stress comparisons with a) without over glue, b) with overglue, c) further wetting of sample and d) a curved cross section profile shape**.

The shear stress is homogeneous inside the joining area, but not constant as it depends on the distance to the centre according to equation 1, being theoretically maximum at the outer radius. Goglio et. al. (7) indicated by FE analysis that an accumulation of stresses at the outer ring edge of an adhesive join could lead to a stress concentration factor of 1.21 to 1.37) on small ceramic specimens (4 and 5 mm join diameter area) with and without holes respectively. Our initial FE analysis on small steel samples without holes, supports these results (Fig 2). However, for larger samples 14 and 20 mm diameter, the effect is drastically reduced (Fig 2), hence our choice of a 14 mm diameter. In addition FE analysis of the angle of the glue (and overglue) has shown that the contact angle between specimen and glue can have a significant but not drastic effect on the shear stress level at the outer surface especially for the smaller specimens (5 mm diameter). In Fig 3A and 3B, samples with zero and a very high contact angle are compared. Wetting of further regions of the specimen (Fig 3C) and a curved cross section profile of the steel specimen (Fig 3D) in comparison have less influence on the maximum shear stress in the glue. These results influenced our choice of specimen dimension, as the larger specimens exhibited lower stresses.

The surfaces of the steel specimens were polished with SiC paper with FEPA #500 mesh. The surface roughness was measured with a Bruker nano profilometer after polishing to ensure that all joining surfaces had a similar roughness. Afterwards the samples were cleaned in acetone in an ultrasonic bath and dried prior to joining.

The Hysol EA9321 epoxy paste adhesive was purchased from Henkel Corporation, CA USA, whilst the 3M Scotch-Weld EC-9323-2 B/A two part structural adhesive was purchased from 3M, Germany. Both adhesives were prepared in accordance to the manufacturers’ instructions. The epoxies were applied on the joining surface and the joints were cured in an alignment jig for 1h at 80°C and 2h at 65°C, for the Hysol and 3M respectively. The thicknesses of the joints were controlled with alumina beads of 200 microns diameter (1 wt. % was added to the adhesive pastes). Any excess adhesive was cleaned from the joining area before curing.

**Fig 4:**
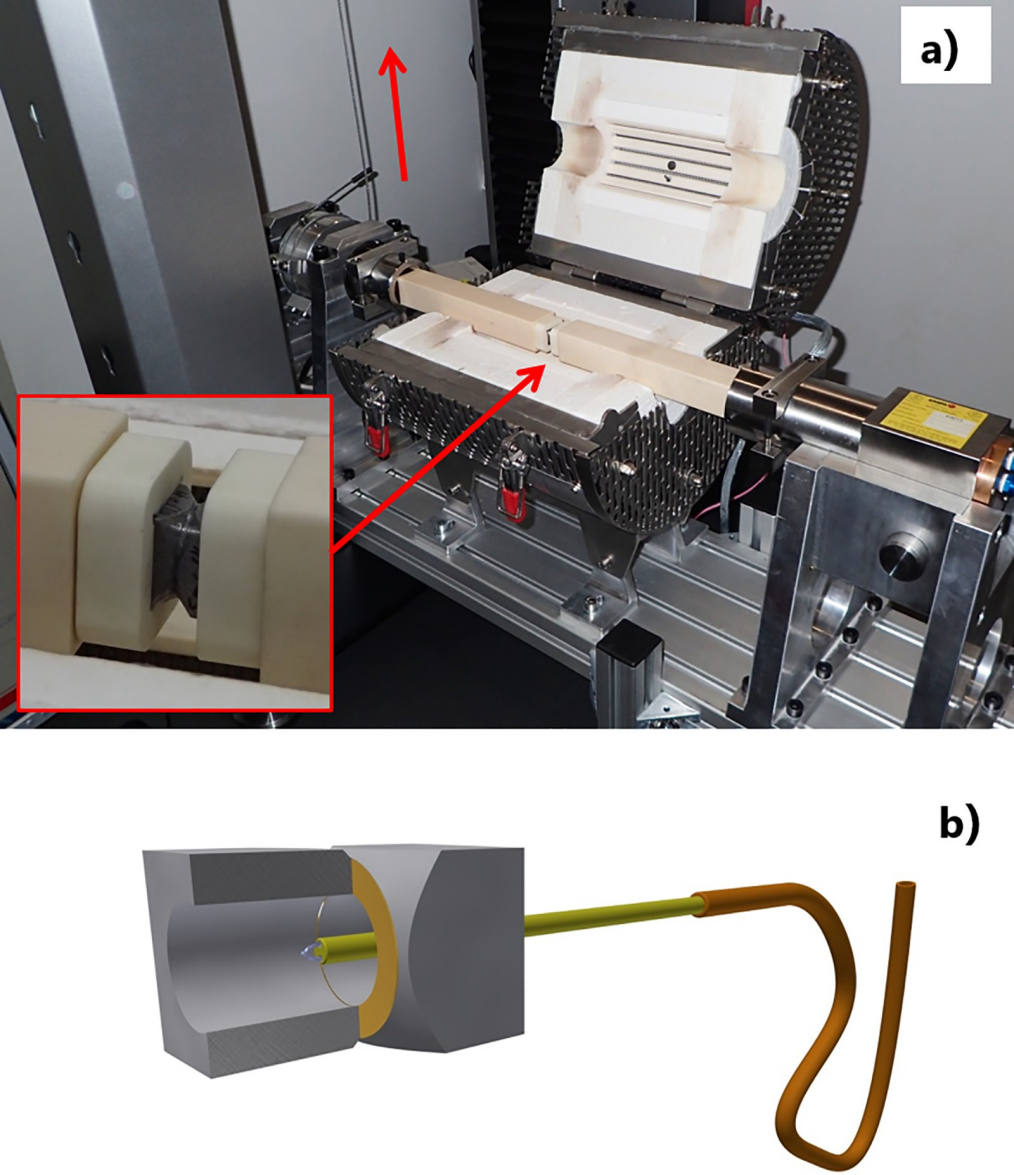
a) The torsional shear test jig in position in the universal test machine with test furnace in position and with alumina arms (close up of test specimen), b) cross section of the test specimen and thermocouple.

**Fig 5:**
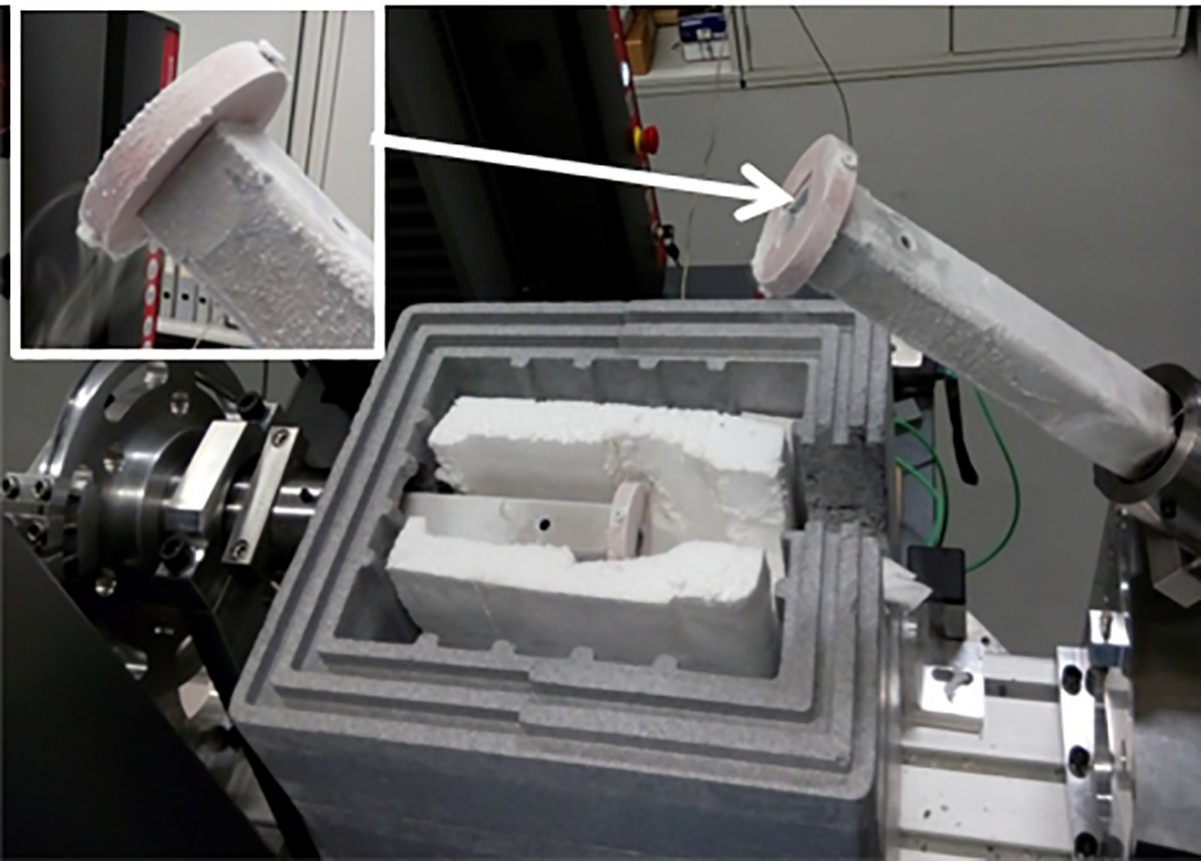
Torsional shear test set up for cryogenic conditions with insulated liquid nitrogen box and copper discs for heat transfer to test specimen.

A torsional shear test machine specially designed and constructed by Empa was used for all testing. This is shown in Fig 4 with a close up of the sample holder. The torsion test jig is driven by a universal test machine (Zwick z005, Zwick GmbH & Co. KG, Germany) connected with a steel pulley cable, the test was performed at a constant crosshead speed of 3 mm/min. The torsional test equipment achieved a Machine Class 1 calibration using a calibrated load cell over the range 40–200 Nm and with cantilever weights from 5–40 Nm in accordance to the DIN Norm 51309. In order to evaluate the torsional shear strength joints at relevant temperatures with respect to different applications, Empa’s torsional test jig is equipped with a furnace capable to 1200°C and an insulated cryogenic box which allows testing at temperatures as low as -190°C. The cryogenic configuration consists of a liquid nitrogen bath in contact with the specimen holders through two large copper plates (see Fig 5). It is important to remark that the samples are not in direct contact with the liquid nitrogen, all the heat transfer is done through the copper plates and the steel arms of the jig. In addition ceramic alumina arms and specimen holders can be used for testing over 500°C. The test temperature was measured with a thermocouple placed through the centre of the torsional arm in close proximity with the internal diameter of the specimen (see Fig 4B). The torsional shear strength *τ_max_* was calculated in MPa in accordance with:

$$\tau_{max}=\frac{Tr_{0}}{I_{p}}$$

Where: $I_{p}=\frac{\pi (r_{OD}^{4}-r_{OI}^{4})}{2}$, is the polar moment of inertia (m^4^), *T* is the applied torque at failure (N m), *r*_0*D*_ = radius to outside full circle of ring as measured (m), where the maximum stress occurs in this case 7 mm, *r*_0*I*_ = radius to inside of ring as measured (m). As the stress in the joining area is dependent on the radius it is straightforward to calculate the shear stress in the middle of the join. The other benefit of the hollow cylinder is to reduce the ductile contribution to the shear strength, however this not really important in the current case.

The coefficient of thermal expansion (CTE) was measured of both adhesives and the steel from -150 to 120°C using a Netsch TMA 402 F3 Hyperion dilatometer (Netsch, Germany). Fractography of the tested specimens was performed using a Zeiss Stereo Discovery V20 optical microscope (Carl Zeiss AG, Germany)

## Results and discussion

### Torsional shear strength

**Fig 6:**
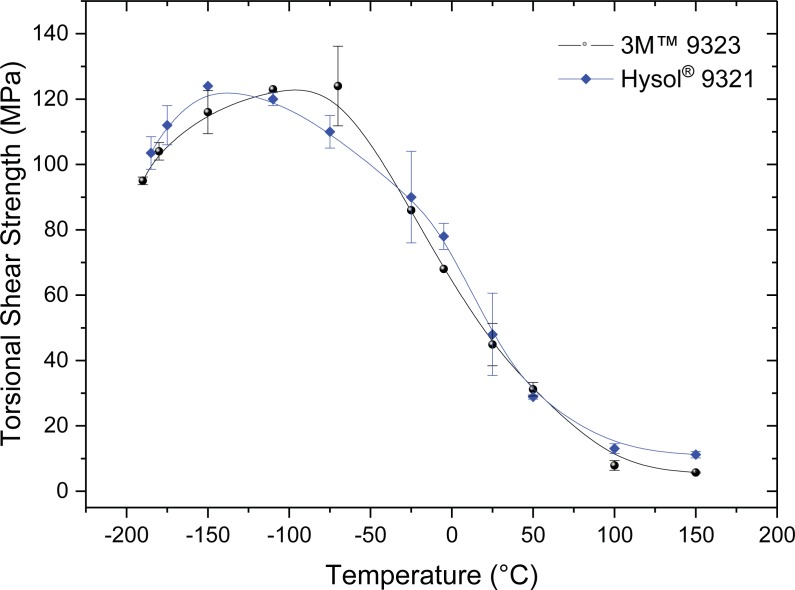
Torsional shear strength at failure versus the corresponding test temperature.

Initial tests were carried out at room temperature, before performing further tests over the temperature range from -180°C to 150°C. Typically at least five measurements were made at each temperature. However, if the adhesive was found not to have covered the whole joining area or there was some excess outside the joint area then the result was discarded. The results of measured torsional shear stress as a function of the test temperature are presented in Fig 6. The results show a surprising and similar trend for both adhesive systems. Both adhesives exhibited maximum torsional shear strengths at cryogenic temperatures in the region of 120 MPa which was approximately 3 times that of their average room temperature torsional shear strengths of 45–50 MPa. The torsional shear strength decreases with increasing test temperature and at 150°C it is 10–15 MPa.

**Fig 7:**
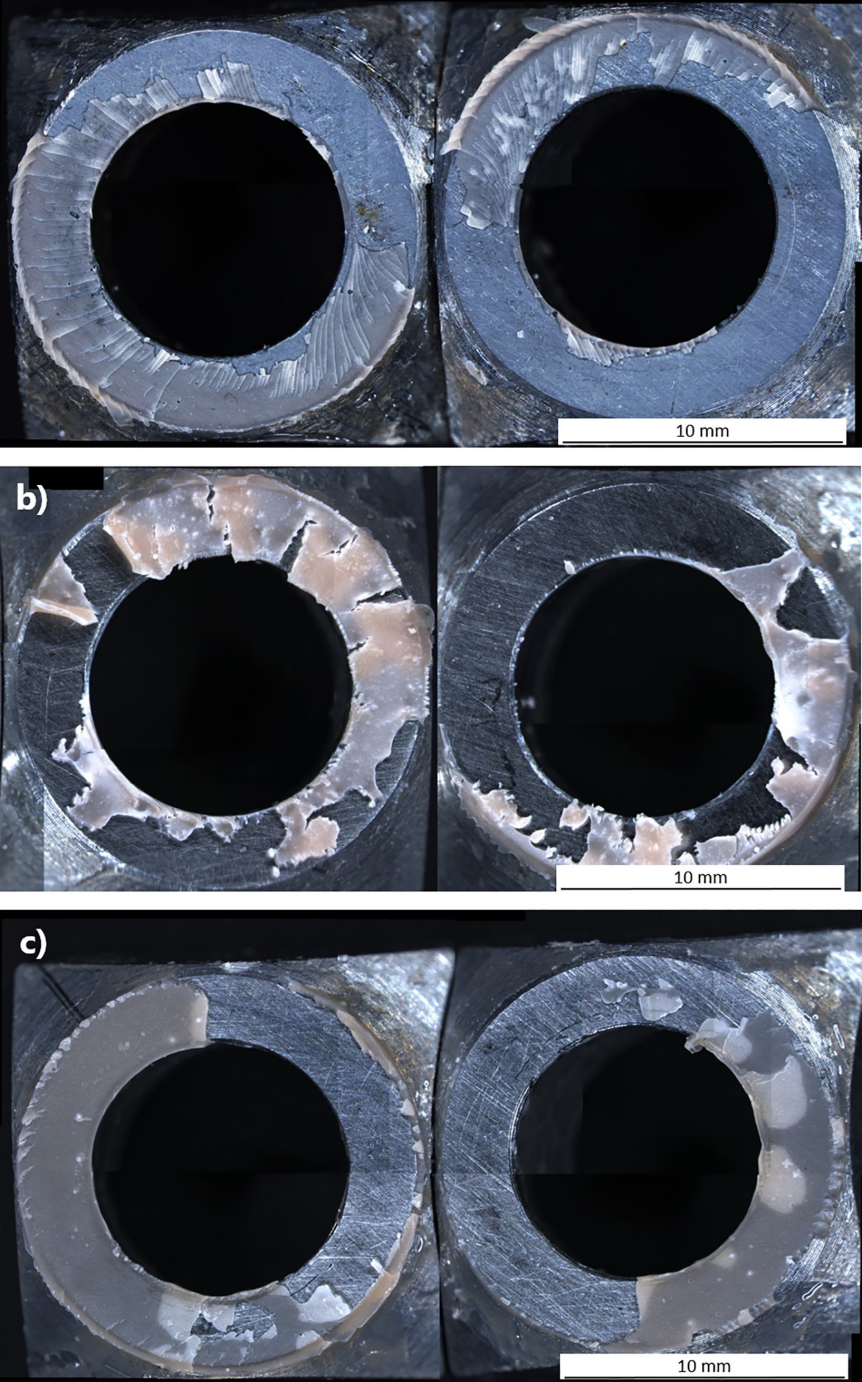
**Failure surfaces of 3M 9323 joints after torsional shear testing**; (a) at -180°C showing many small cracks in the adhesive formed when it was in a brittle state, (b) failure surfaces of 3M 9323 joints after torsional shear testing at 25°C showing ductile tearing and ductile peeling of the adhesive, (c) failure surfaces of 3 9323 M joints after torsional shear testing at 100°C showing failure with limited tearing and some partial peeling from the steel substrate.

Analysis of the fracture surfaces of the specimens was performed to try to understand the reasons behind these differences in strength, especially under cryogenic conditions. The fracture surfaces showed that although failure always occurred through the adhesive substrate layer as expected, that different types of failure occurred in the adhesive at the different test temperatures. Steel joined with 3M is used to illustrate the differences at the testing temperatures in Fig 7, in all cases both fracture surfaces are exhibited. The 3M steel joint fracture surfaces are shown from samples tested at -180°C, 25°C and at 100°C in Fig 7A, 7B and 7C respectively. In Fig 7A the fracture surface of a sample from -180°C shows a brittle type of failure in the adhesive, where large areas of the adhesive are still intact. However, there are many small cracks which are present in the intact adhesive (see magnified insert). The increase in stress in the “brittle” adhesive is due to it becoming harder and stiffer with the decrease in temperature leading to these cracks in the white non-opaque adhesive. These cracks show that the high stresses were spread uniformly around the entire load area, resulting in cracks in the adhesive around the diameter of the joint. The cracks appear to grow from the inner diameter towards the outer diameter, as many cracks are visible extending from the inner diameter but have not reached the outer diameter (see arrow in Fig 7A). The reasons for the cracks appearing to grow from the inner to the outer diameter is not clear. In addition the uniform location of these microcracks around the adhesive joint indicates that the shear stress field is uniform and no stress concentrations due to the test setup or specimen geometry are present. The failure surfaces of a 25°C sample in Fig 7B show a more ductile type of peeling of the adhesive from the steel substrate as well as tearing of the adhesive. The tearing appears also to be ductile in nature. The failure surfaces of a sample tested at 100°C are shown in Fig 7C, the failure appears to occur by peeling with a much reduced number of tear points compared to 25°C. There are also regions visible where separation of the adhesive layer from the substrate has started but not reached a critical state so as to separate from the steel substrate. This indicates an increased ductile behaviour of the adhesive with increasing test temperature. At the higher temperatures it is not possible to confirm that the failure starts from the inner diameter.

**Fig 8:**
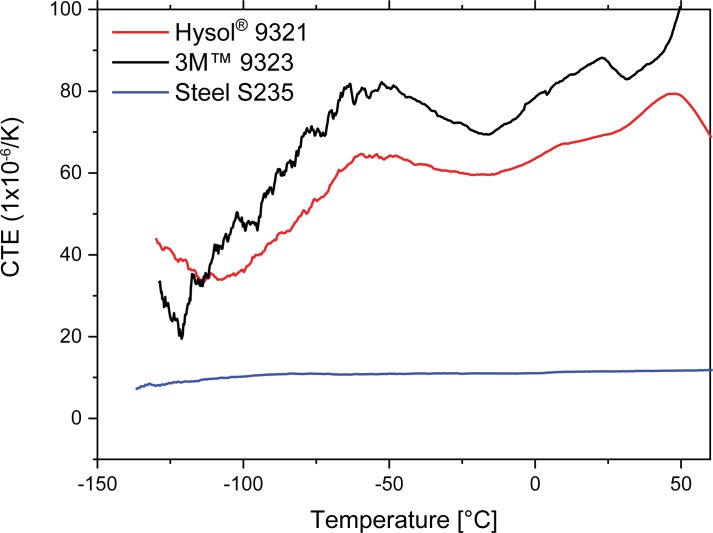
Coefficient of thermal expansion results of the different joint materials Hysol9321, 3M9323 and S235 steel used in the test joints.

**Fig 9:**
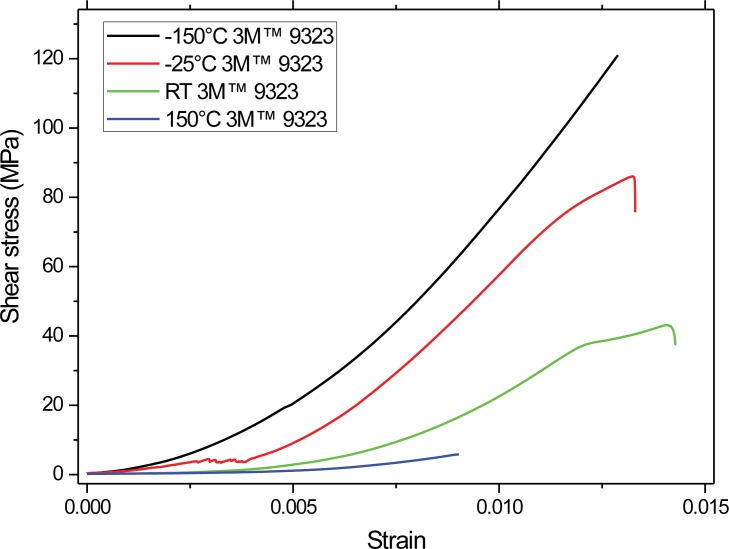
Torsional shear stress vs strain for the 3M 9323 joined steel samples at different test temperatures, showing increase in stiffness with lower test temperature.

The CTE measurements of the bulk materials are shown in Fig 8 from a temperature range of -150°C to 120°C. The CTE of the steel is pretty consistent in the temperature range of the testing. Whilst the CTEs of both adhesives increase as a function of temperature. Increased CTE mismatch between the substrate and the adhesive is believed to result in increased thermal mismatch stresses including normal residual stresses but in the current instance with increasing temperature the softening of the adhesives is most likely to be the most prevalent effect. Of course the purpose of using the torsional shear set up is that it should avoid normal stresses, and it has already been shown that increasing temperature results in a relaxation of residual stresses in joins with CTE mismatches [13]. The calculated stress vs strain curves of samples joined with 3M at four different test temperatures are shown in Fig 9, the initial region shows the taking up of slack in the machine. The strain was calculated from the cross-head displacement of the test machine. The shear strain is as defined by the mechanics of materials. Of interest are the linear regions after strain of 0.005, which clearly show that the stiffness increases with temperature, resulting in a brittle almost glass like failure at -150°C. This is in complete agreement with our observations of the fracture surfaces. The Hysol has Tg of 110°C whilst the 3M EC-9323 has Tg of 94°C. The strain at under cryogenic conditions is much greater than at RT and 150°C, for example at 20 MPa shear stress this is over 1% from -150°C to RT, this is similar to results from Huveners et. al. who tested epoxy-aluminium joins from -20°C to 80°C, and observed under freezing conditions that epoxy has larger ultimate shear stresses than at 23°C, but behaves brittle[14]. Whilst temperatures larger than the glass transition temperature led to significantly smaller values for the ultimate shear strength of their joints. In addition work by da Silva on epoxy adhesives in tensile and shear (TAST) showed similar strain to our results[15].

There is very limited shear test data of adhesively bonded joints under cryogenic test conditions. In the work by Kang e.t. al. of three different types of adhesives used to join aluminium and tested in double lap joint configuration at 25°C and -150°C all three systems showed a much higher strength at -150°C than at RT [11]. With the best improvement nearly doubling the measured joint strength from 25.1 to 47.7 MPa. Kang et. al. believed the observed increase was due to an increase in the stiffness of the epoxy adhesives in the joint with decreasing temperature. The double lap shear strength measurements of Kang at -150°C [15]are still well below our highest measured torsional shear strength values of approximately 120 MPa for both adhesives in cryogenic conditions. This might be due to us measuring pure shear in torsion, whereas Kang measured in double lap configuration.

Torsional shear tests were also performed at room temperature on epoxy-stainless steel joints of “hour glass” type samples of various size and fillet size by Jung et.al. [16]. However, the significant scatter of the three different geometries tested was so high, e.g. for one specimen dimension the average torsional shear strength was 17.5 MPa (with a minimum at of 8 MPa and maximum of 32 MPa) that the comparison of the different specimen geometries and dimensions is difficult. This large scatter may in fact also be due to the preparation, as we have determined it is important to prepare all test specimens carefully with a similar bond thickness and no excess of adhesive coming out of the joint area. Jung et. al. gave no details on the preparation or even the grades of steel or epoxy used. In another research by Murakami et. al. on epoxy bonded steel butt joints had higher values but the testing was combined torsional shear and tensile testing together and can not be compared directly with our pure torsional shear test [17].

### Torsional shear fatigue and cryogenic aging

**Fig 10:**
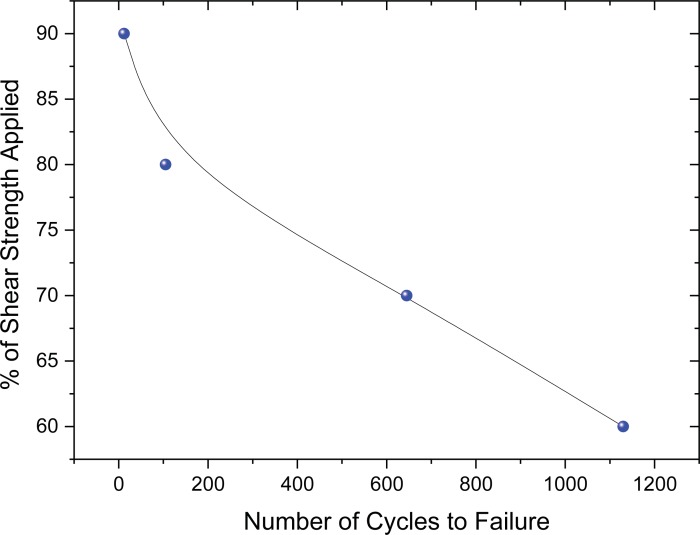
Room temperature torsional shear fatigue test of the Hysol9321 steel joints to show the effect different torsional shear strength load (% of failure strength) versus the number of cycles to failure.

Torsional shear fatigue tests were performed at room temperature on Hysol steel joints at various loads. At room temperature the average torsional shear strength was 47 MPa. Preliminary tests were performed at 50% of this load and samples did not fail even, after 100,000 cycles. Thereafter, fatigue tests were performed at different loads at 90, 80, 70 and 60% of the average shear strength. The fatigue test was performed at a cyclic frequency of 0.5 Hz. The results are presented in Fig 10. As can be expected the number of cycles to failure is clearly increasing with decreasing load. However, with our results it is difficult to be able to determine if there is a direct mathematical correlation fitting to the change in behaviour observed, this is also in part due to the limited number of specimens tested (one at each load). Other effects such as the presence of voids or bubbles in the adhesive can be even more critical in this type of test than in direct torsional shear strength measurements as these defects can cause initialization of critical failure origins with time. This testing was carried out as a feasibility study to assess the suitability of the torsional shear test equipment for performing shear fatigue, the results indicate it is a useful tool for making these measurements.

**Fig 11:**
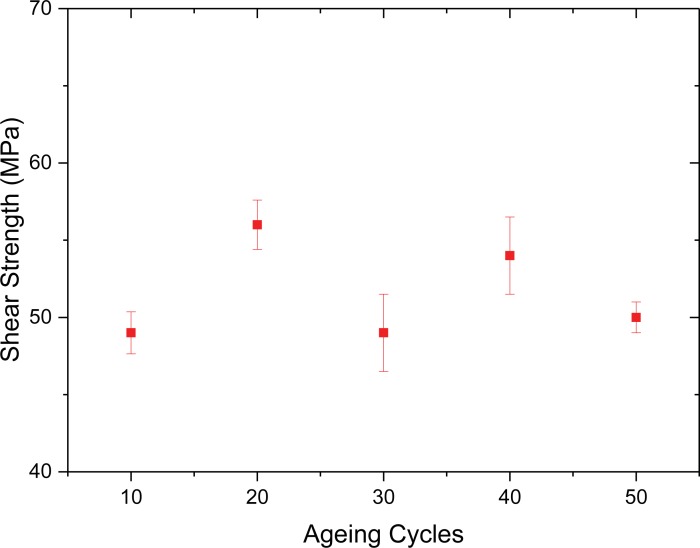
Shear strength tests after cryogenical aging of Hysol steel joints from -180°C to RT and the measured strength after 10, 20, 30, 40 and 50 such cycles.

Thermal cyclic cryogenic aging tests were also performed on Hysol steel samples. The specimens were cryogenically cooled in liquid nitrogen to -180°C and then naturally warmed again to RT. This was repeated for an increasing number of such cycles (10, 20, 30, 40, and 50) before the specimens were then tested for torsional shear strength at room temperature. The results presented in Fig 11 show that with up to 50 of such aging cycles there is no significant change in the measured torsional shear strength. This might be due to the relatively slow nature of warming from -180 to RT not creating any significant microcracks in the adhesive and/or peeling due to the different CTEs of the steel and the Hysol. Another possibility is that the plasticity of the adhesives may help to prevent any microcracks from growing during cooling and heating. The average strength is similar to that measured for Hysol steel joints which have not undergone any such thermal aging cycles.

## Conclusions

We have developed and verified a torsional shear setup using model steel-epoxy adhesive systems allowing the measurement of pure shear. We can conclude that:

- The setup functions well over a wide temperature range at cryogenic and elevated temperatures. The use of test samples with a hole allows the measurement of the test temperature by locating a thermocouple precisely inside the test sample.
- The torsional shear strength of both types of adhesives increases with decreasing temperature and is believed to be associated with the change in stiffness with decreasing temperature. Both adhesives (3M and Hysol) were found to change behaviour from brittle to ductile failure with increasing temperature from -180°C to 150°C.
- In addition the torsional shear strength measured (120 MPa) under cryogenic conditions is higher than any published for similar adhesive systems for joining to metal.
- The test equipment was also found to be very suitable for measurement of cyclic fatigue at room temperature.

For the future outlook we will be performing cyclic fatigue at different temperatures (cryogenic and elevated) and of different joint systems including brazed ceramic to ceramic joints and ceramic to metal joints. We have already published torsional shear results of brazed ceramic joints with very high strengths from RT to 400°C [8] and can foresee testing of appropriate joining systems at even higher temperatures as well as under cryogenic conditions. It is our aim that this test becomes more adapted to provide designers and engineers with more reliable shear strength data when designing joined components.
